# The effect of elevated temperatures on the life history and insecticide resistance phenotype of the major malaria vector *Anopheles arabiensis* (Diptera: Culicidae)

**DOI:** 10.1186/s12936-017-1720-4

**Published:** 2017-02-14

**Authors:** Shüné V. Oliver, Basil D. Brooke

**Affiliations:** 1 Centre for Opportunistic, Tropical and Hospital Infections, National Institute for Communicable Diseases, 1 Modderfontein Road, Sandringham, Johannesburg, South Africa; 2 Faculty of Health Sciences, Wits Research Institute for Malaria, School of Pathology, University of the Witwatersrand, 7 York Road, Parktown, Johannesburg, South Africa

**Keywords:** Thermal stress, Larval development rate, Adult longevity, Insecticide resistance, Cross-tolerance, Heat shock protein

## Abstract

**Background:**

Temperature plays a crucial role in the life history of insects. Recent climate change research has highlighted the importance of elevated temperature on malaria vector distribution. This study aims to examine the role of elevated temperatures on epidemiologically important life-history traits in the major malaria vector, *Anopheles arabiensis*. Specifically, the differential effects of temperature on insecticide-resistant and susceptible strains were examined.

**Methods:**

Two laboratory strains of *A. arabiensis*, the insecticide-susceptible SENN and the insecticide-resistant SENN DDT strains, were used to examine the effect of elevated temperatures on larval development and adult longevity. The effects of various elevated temperatures on insecticide resistance phenotypes were also examined and the biochemical basis of the changes in insecticide resistance phenotype was assessed.

**Results:**

SENN and SENN DDT larvae developed at similar rates at elevated temperatures. SENN DDT adult survivorship did not vary between control and elevated temperatures, while the longevity of SENN adults at constantly elevated temperatures was significantly reduced. SENN DDT adults lived significantly longer than SENN at constantly elevated temperatures. Elevated rearing temperatures, as well as a short-term exposure to 37 and 39 °C as adults, augmented pyrethroid resistance in adult SENN DDT, and increased pyrethroid tolerance in SENN. Detoxification enzyme activity was not implicated in this phenotypic effect. Quercertin-induced synergism of inducible heat shock proteins negated this temperature-mediated augmentation of pyrethroid resistance.

**Conclusion:**

Insecticide-resistant *A. arabiensis* live longer than their susceptible counterparts at elevated temperatures. Exposure to heat shock augments pyrethroid resistance in both resistant and susceptible strains. This response is potentially mediated by inducible heat shock proteins.

## Background

Exposure to high temperatures has a variety of physiological effects on poikilothermic animals such as insects. These effects include increases in metabolic rate [1, 2] and modifications of biochemical processes [3]. Aquatic insects, or the aquatic immature stages of insects such as mosquitoes, are particularly affected by temperature due to the high thermal conductivity of water as well as the limited ability of these insects to escape adverse temperatures [4]. In many insects the environmental conditions experienced during the aquatic stages affect larval biology. For example, assessments of the effects of temperature on the major malaria vector *Anopheles gambiae* showed that larval development rate tends to increase as temperature increases [5]. High temperatures can also affect the life-history parameters of adult *Anopheles* mosquitoes. For example, young adult mosquitoes cope better with elevated temperatures than those of older age groups [6] and elevated temperatures have been shown to decrease the lifespan of some insects [7]. Consistent with the increase in metabolic activity under conditions of increased temperature, several antimicrobial peptides are overexpressed at high temperatures in *Aedes aegypti* mosquitoes [8].

Fluctuating temperatures, particularly extreme high and low temperatures, have the potential to impact insecticide-based malaria control interventions through their effects on the target mosquito vector populations [9]. This is due to two factors. The first is the variable activity of insecticides at different temperatures whereby some insecticides show higher toxicity at higher temperatures (positive temperature coefficient) while others are less toxic at higher temperatures (negative temperature coefficient). Temperature coefficients vary by insecticides and species [10] and have most recently been examined in *A. gambiae* [11]. Secondly, temperature directly alters the physiology of mosquitoes. Simultaneous exposure to elevated temperatures and insecticides have been demonstrated to induce cross-tolerance in anopheline larvae [12]. Furthermore, certain insecticide resistance phenotypes have been associated with thermal tolerance. Malathion-resistant *Culex quinquefasciatus* have been shown to be more tolerant of high temperatures than their susceptible counterparts [13]. Elevated temperatures have also been shown to augment pyrethroid resistance in *C. quinquefasciatus* [14], increasing α-esterase activity while decreasing β-esterase activity [15].

Most studies on elevated temperature and its effect on malaria transmission have focussed primarily on changing distribution of vectors, rather than on the effects on mosquito physiology. Until recently, very little research has been conducted on the effects of temperature on mosquito physiology that may have epidemiologically significant consequences (see [16, 17]).

*Anopheles arabiensis*, a major malaria vector in southern Africa, is well adapted to hot, arid conditions [18, 19] and has been shown to cope better with fluctuating temperatures than other sympatric vector species such as *Anopheles funestus* [20]. The variable resting and feeding behaviour of *A. arabiensis* renders this species less susceptible to control by indoor insecticide application because adult females will often feed and rest outdoors (exophily) [21, 22]. Given that *A. arabiensis* is generally more difficult to control than other vector species with stronger tendencies to feed and rest indoors (endophily), it is important to assess whether environmental conditions can also affect the biology and control of this species. The aim of this study was to assess the effects of elevated temperatures on the life history and insecticide resistance phenotypes of laboratory-reared strains of *A. arabiensis*. Elevated temperature, rather than fluctuating temperature, was used to examine a proof-of-principle concept about the response to a single environmental stressor, rather than trying to mimic the variable conditions of natural environments.

## Methods

### *Anopheles* strains used in the experiments

Two laboratory strains of *A. arabiensis* were used in this study:

- SENN—colonized from Sennar in Sudan in 1980. This strain currently shows low-level permethrin resistance, but is fully susceptible to all other insecticides.
- SENN DDT—intensively selected from SENN for resistance to DDT. SENN DDT currently shows resistance to DDT, malathion, deltamethrin, permethrin, and λ-cyhalothrin. Resistance in this strain is mediated by elevated cytochrome P450, GST and esterase activity. The strain is also fixed for the L1014F *kdr* mutation [23, 24]. Larvae were reared as per Hunt et al. [25]. Deviations from this procedure are detailed by experiment.

### The effect of elevated temperature on larval development

To determine the effect of elevated temperature on development, groups of 25 first instar SENN and SENN DDT larvae (within 24 h after hatching) were placed into 1000 ml of distilled water. Each group was then incubated at one of three temperatures: the control group was kept at standard insectary conditions of 25 °C and 80 ± 5% relative humidity [25]; the first experimental group was raised in an insectary set at 30 ± 2 °C and 80 ± 5% relative humidity; the second experimental group was raised in an incubator set at 35 °C, with additional water bowls used to raise the humidity in the incubator to 80 ± 5%. A free supply of air into the incubator was ensured at all times. The 1000-ml water volume for each group was constantly maintained for all treatments for the duration of the experiment. The experiment was replicated from separate cohorts three times. For each cohort, five biological replicates were used for the control and temperature (30 and 35 °C) treatments. The time to pupation was monitored as a measure of development time.

### The effect of elevated temperature on adult longevity

To determine the effect of elevated temperature on longevity, SENN and SENN DDT first instar larvae were used as described for the previous experiment, but only at the control temperature of 25 °C and the elevated temperature of 30 °C. For each strain, four bowls of 30 larvae each were incubated for each of three experimental replicates at both the control temperature and the experimental temperature of 30 °C. Adults that emerged were placed in cages with ad libitum access to sugar water, but were not allowed to mate or blood-feed.

The adults that emerged from rearing at the control temperature (25 °C, standard insectary conditions) were immediately split into two groups. The first group remained at 25 °C, while the second group was moved to the elevated temperature of 30 °C. Similarly, for the larvae that were reared at 30 °C, adults were separated upon emergence with half remaining at 30 °C, while the other half were moved to standard insectary conditions with a temperature of 25 °C. Each group then remained at the temperature that they were moved to for the duration of the experiment. This means that a 25 °C reared group spent the experiment at either 25 or 30 °C, and a 30 °C reared group spent the experiment at either 25 or 30 °C. Adult longevity was monitored as a function of survival daily, with cadavers removed on the day of death. The 10% sucrose solution offered to each cohort was refreshed on a daily basis.

### The effect of elevated larval-rearing temperature on the subsequent expression of adult insecticide resistance phenotypes

To determine the effect of elevated larval-rearing temperatures on subsequent adult insecticide resistance phenotype, SENN DDT larvae were incubated at 30 °C, with control larvae incubated at 25 °C, starting within 24 h of hatching. Equal numbers of larvae were kept in 1000 ml of distilled water and were fed an equal amount of larval food three times daily. Upon emergence, adults from the control and treatment cohorts were collected and kept at 25 °C with ad libitum access to 10% sucrose, but without any access to blood until they were 3 days old. At the age of 3 days the treatment cohorts were exposed to either 5% malathion (♂: n = 367; ♀: n = 450) or 0.05% deltamethrin (♂: n = 380; ♀: n = 435) using the standard WHO insecticide susceptibility procedure [26]. Similarly, control (unexposed to elevated temperatures) adult male and female mosquitoes were exposed to malathion (♂: n = 342; ♀: n = 444) and deltamethrin (♂: n = 464; ♀: n = 582). This experiment was replicated three times from three separate cohorts. Mortality was scored 24 h post exposure. A control cohort exposed to untreated paper only constituted a handling control, while a completely unexposed group constituted an environmental control. All handling and environmental controls were drawn from the same populations that were being exposed to the insecticides, i.e., when exposing adults emerging from larvae reared at elevated temperatures, their accompanying handling and environmental controls would come from the same cohort of adults. Similarly, when exposing adults with no exposure to elevated temperatures, these adults would constitute the handling and environmental controls.

### The effect of short-term heat shock on the expression of the adult insecticide resistance phenotypes

Mosquitoes may be exposed to short-term heat shock during the day, or during a blood meal. To determine the effect of a short-term heat shock on the insecticide resistance phenotype of SENN DDT, larvae were reared under standard insectary conditions, and then the adults were exposed to a short-term heat shock at 3 days of age. For the first treatment, a mixture of 3-day old adult males and females were exposed to a heat shock of 37 °C for 3 h. During this time they were not allowed access to sugar. Immediately after the shock period they were allowed to recover for an hour with access to sugar. The heat-shocked adults were then exposed to either 5% malathion (♂: n = 394; ♀: n = 489) or 0.05% deltamethrin (♂: n = 349; ♀: n = 408) using the standard WHO insecticide susceptibility procedure [26]. A second experimental SENN DDT group was exposed to the sub-lethal heat-shock temperature of 39 °C for 1 h, before exposures to malathion (♂: n = 412; ♀: n = 423) or deltamethrin (♂: n = 370; ♀: n = 412). The mortalities of the heat-exposed adults were compared to adults not exposed to elevated temperature [malathion: (♂: n = 425; ♀: n = 473); deltamethrin (♂: n = 395; ♀: n = 412)]. A control cohort exposed to untreated paper only constituted a handling control, while a completely unexposed group constituted an environmental control. All handling and environmental controls were drawn from the same populations that were being exposed to the insecticides, i.e., when exposing heat-shocked adults, their accompanying handling and environmental controls would come from the same cohort of adults. Similarly, when exposing adults with no exposure to elevated temperatures, these adults would constitute the handling and environmental controls.

### The lasting effects of short-term heat shock on the expression of the adult insecticide resistance phenotypes

A set of experiments were conducted to determine whether the effects of short-term heat shock changed with time. For these experiments, only the pyrethroids deltamethrin and λ-cyhalothrin were used, and only adult females were assayed. A 3-day old, non-blood-fed cohort of SENN DDT, reared at standard insectary temperature, was exposed to either 37 °C for 3 h or 39 °C for 1 h. After a 1-h recovery period, standard WHO bioassays using 0.05% deltamethrin were performed every hour from 2 to 7 h post shock for both heat shock temperature cohorts (37 and 39 °C): 37 °C control, n = 320; 2 h: n = 353; 3 h: n = 343; 4 h: n = 361; 5 h: n = 320; 6 h: n = 317; 7 h: n = 309; 39 °C control, n = 330; 2 h: n = 362; 3 h: n = 370; 4 h: n = 351; 5 h: n = 343; 6 h: n = 330; 7 h: n = 321. The exposed control group was constituted of a group of SENN DDT from the same batch of adults that had not been exposed to a heat shock. Each experiment was replicated three times using three different cohorts. As deltamethrin resistance expression was affected by heat shock temperature (as demonstrated in Fig. 4A), another pyrethroid, 0.05% λ-cyhalothrin was also assayed after a 3-h 37 °C shock with a 1-h recovery period and 1-h 39 °C exposure followed by a 1-h recovery period (37 °C control: n = 220; 2 h: n = 253; 3 h: n = 249) (39 °C control: n = 230; 2 h: n = 242; 3 h: n = 277).

To ensure that sugar deprivation did not affect the results, another control group not exposed to a heat shock but deprived of sugar for the same period of time as their heat-shocked counterparts was used. Therefore, control exposures as well as unexposed controls included groups of individuals that were not exposed to elevated temperatures (n = 111), but half were allowed sugar for the duration of their exposure period (n = 117), and the other half were deprived of sugar in the same manner as the heat-exposed groups (n = 139).

### The effect of short-term heat shock on insecticide susceptibility in an insecticide susceptible strain

The effect of short-term heat shock on the insecticide susceptibility phenotype of the SENN strain was examined using 3-day old non-blood-fed SENN adults that were exposed to either a 3-h 37 °C heat shock or a 1-h 39 °C heat shock. After a recovery period of 1 h, ten replicates of 20–25 females each were exposed to either 0.75% permethrin (n = 523), 0.05% deltamethrin (n = 473) or 0.05% λ-cyhalothrin (n = 492) using the standard WHO susceptibility methods [26]. Non-heat-shocked individuals were exposed to the same insecticide treatments concurrently. Individuals from the heat-shocked and non-heat-shocked groups were exposed to untreated papers, and this served as a handling control. Individuals from the heat-shocked and non-heated-shocked groups that were not exposed to any paper (treated or untreated) served as environmental controls.

All short-term heat shock treatments were performed by exposing the adults to the required temperature in an incubator in which the humidity had been raised to ±80% humidity by the addition of a water source. All insecticide exposures were performed at 25 °C as per standard WHO bioassay conditions [26].

### The effects of elevated temperatures on detoxification enzyme activity

As heat shock exerted the strongest effect on pyrethroid resistance expression, the effect of temperature on cytochrome P450 activity and general esterases was examined as these enzymes are mediators of pyrethroid resistance [27] and are not associated with the *kdr* mutation.

Two temperature assays were performed. Standard haem peroxidase and general esterase activity assays were performed on 96 male and 96 female SENN DDT adults [28, 29]. For each individual a duplicate reaction plate was prepared. Once all of the reagents were added, the control plate was allowed to incubate at 25 °C (control reaction) while the experimental reaction was allowed to incubate at 30 °C. After the requisite incubation periods the amount of product formed was determined by measuring the absorbance at 650 nm for the haem peroxidase assay and 570 nm for the esterase assay. This experiment was replicated three times, with adults for each replicate being drawn from a new cohort, i.e., siblings from a new egg batch each time.

The second experiment was designed to determine whether adult SENN DDT had altered cytochrome P450 activity after heat shock. For this experiment, 96 SENN DDT individuals (48 males and 48 females), were drawn from replicates that had been exposed to 39 °C for 1 h, but without any exposure to insecticides. Adults not exposed to heat shock constituted the control group. As for the previous experiments, the heat-shocked group was not allowed access to sugar for their period of heat shock. At the same time, the control group was also deprived of sugar. Forty-eight adults of each gender were drawn from the shocked and control groups at 1 h intervals after heat shock, up to the age of 7 h. These individuals were cold-killed and immediately stored at −70 °C, after which they were processed within a week for haem peroxidase and esterase activity as described above.

### The role of inducible heat shock proteins in the insecticide resistance phenotypes

To determine whether heat shock proteins (HSPs) played a role in the effect of elevated temperatures on the expression of the insecticide resistance phenotype, an HSP synergist assay was performed. The flavinoid quercetin was successfully used as a dietary synergist in *Drosophila melanogaster* [30], so a similar assay, using sugar as a delivery mechanism, was developed based on those methods [30]. Five cages of 3-day old SENN DDT adults were prepared using equal numbers of adults (150 males + 150 females) in each. Two of the cages then had their sugar removed and were exposed to a 3-h, 37 °C heat shock. Three cages remained at 25 °C: one had the sugar removed for the 3-h period; the second was supplied with a 10% sucrose solution supplemented with 25 mM quercetin; and the third had full access to sugar solution. The experiment was replicated in triplicate.

After the 3-h heat shock, the heat-stressed mosquitoes were immediately returned to 25 °C and allowed to recover for 2 h with either plain sucrose solution or quercetin-supplemented sucrose. The cage deprived of sugar at room temperature was split into two groups, one of which was supplied with sucrose solution and the other supplied with quercetin-supplemented sucrose. After recovery, the sucrose/quercetin-treated and sucrose-treated individuals were exposed to either 0.05% deltamethrin (treated: ♂: n = 373; ♀: n = 417; control ♂: n = 389; ♀: n = 397) or 0.05% malathion (treated: ♂: n = 354; ♀: n = 423; control ♂: n = 409; ♀: n = 417) using the standard WHO bioassay method [26].

Four controls that were unexposed to insecticide were used for this experiment: a standard environmental control, a control to ensure that quercetin supplementation after starvation did not affect mortality (no heat − sugar + quercetin, n = 153), a control to ensure that quercetin supplementation alone did not elicit mortality (no heat + quercetin, n = 161), and a control to ensure that the 3-h period without sugar did not elicit mortality (no heat − sugar, n = 157).

### Data analysis

All data analysis was performed using Statistix 8 (Talahassee, FL, USA). All analyses were performed at 95% confidence. Means were compared using either a two-sample t test or a one-way analysis of variance (ANOVA) with a Tukey HSD as a post hoc test. Longevity was assessed using a Kaplan–Meier estimator, with a Log-rank test used as a measure of significance.

## Results

### The effects of elevated temperature on larval development rate and adult longevity

**Fig. 1:**
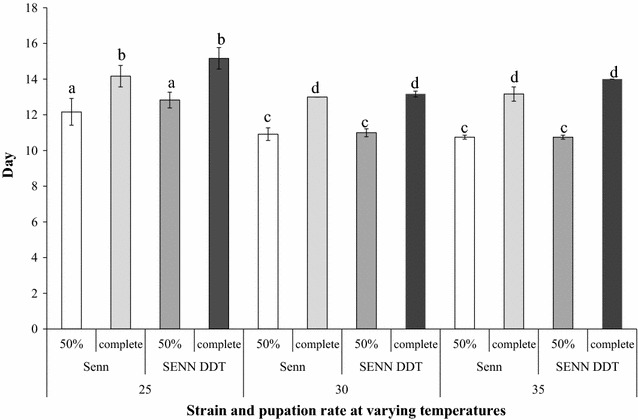
The effects of constant temperature elevation on insecticide resistant and susceptible *Anopheles arabiensis.* Although elevated temperatures decreased time to pupation [measured as time taken for 50% of the population to pupate (50%) and to complete pupation], there were no significant differences in development rate between the susceptible (SENN) and resistant (SENN DDT) strains. Development rate at 30 and 35 °C did not differ significantly in either of the strains. Development rates that do not differ significantly are denoted by the *same letter*

Constantly elevated temperature had a variable effect on larval and adult life history. Although elevated temperatures significantly increased the rate of larval development in SENN DDT (one-way ANOVA: p < 0.01; F = 15.1) and SENN (one-way ANOVA: p < 0.01; F = 12.4) compared to their respective 25 °C control cohorts, there was no significant difference in development rate between 30 and 35 °C (one-way ANOVA: p = 0.34; F = 2.37) for either strain. Furthermore, there was no significant difference in development rate between the two strains at any of the incubation temperatures (one-way ANOVA: 25 °C—p = 0.46; F = 0.59; 30 °C—p = 0.84; F = 0.04; 35 °C—p = 0.08; F = 0.2) (Fig. 1).

**Fig. 2:**
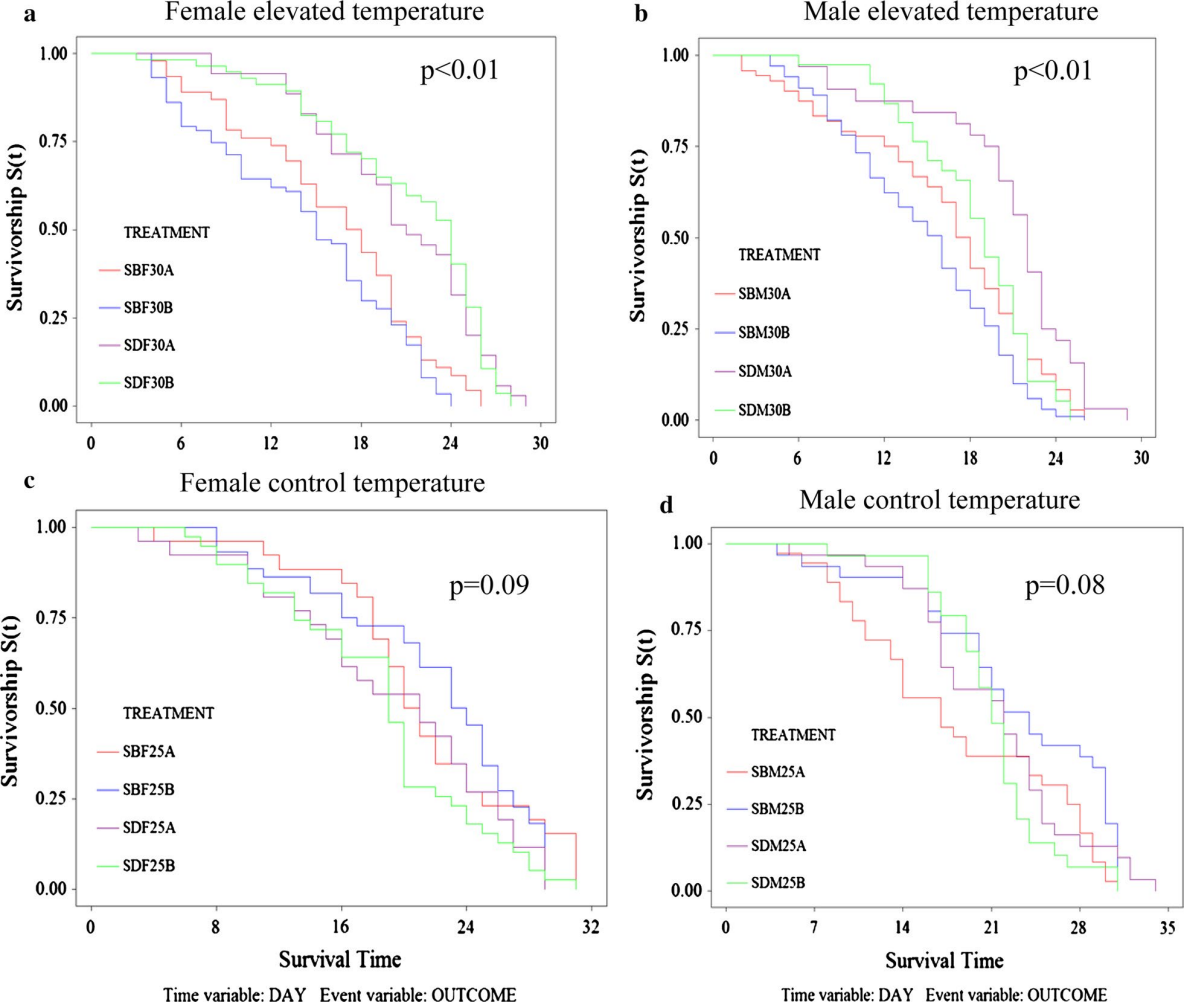
The effects of constant temperature elevation on the longevity of *Anopheles arabiensis* adults. Adults of the resistant SENN DDT strain lived significantly longer when exposed to a constantly elevated temperature of 30 °C. This was true for adult females that emerged from larvae that were reared at 30 °C (SDFA) as well as those reared at 25 °C and only taken to 30 °C upon emergence (SDFB) (**a**). This was also true for SENN DDT males reared as larvae at 30 °C (SDMA) compared to their SENN counterparts (SBMA) as well as DDT males reared at 25 °C as larvae and then incubated at 30 °C upon emergence (SDMB) compared to their SENN counterparts (SBMB) (**b**). There were no significant differences in female longevity of the strains reared at 25 °C (**c**), as well as in male longevity (**d**), regardless of larval-rearing temperature

SENN DDT adult females (Fig. 2a) and males (Fig. 2b) lived significantly longer than their SENN counterparts, regardless of whether the adults emerged from larvae that were reared at 25 °C (Treatment A) or 30 °C (Treatment B) (females log rank test; p < 0.01; χ^2^ = 5.28; males log rank test p < 0.01; χ^2^ = 1.12). There was no difference in longevity between SENN and SENN DDT adults incubated at 25 °C regardless of whether they were raised as larvae at 25 or 30 °C (females log rank test; p = 0.81; χ^2^ = 2.97; males log rank test p = 0.80; χ^2^ = 1.07) (Fig. 2c, d, respectively). SENN adults lived for a significantly shorter period at elevated temperatures compared to control temperatures (females: log rank test p = 0.01, χ^2^ = 6.86, df = 1; males: log rank test: p < 0.01, χ^2^ = 11.95, df = 1), while SENN DDT adult longevity did not differ significantly between the two temperatures (females: log rank test p = 0.25, χ^2^ = 1.34, df = 1; males: log rank test: p = 0.86, χ^2^ = 0.27, df = 1). The time taken to achieve 50% mortality (LT50) by strain and gender is summarized in Table 1.

**Table 1:** Time taken to induce 50% mortality by larval and adult temperature regime by strain and gender

Temperature (^o^C)	SENN ♀	Strain [time to 50% mortality (95% CI)]		SENN DDT ♂
		SENN ♂	SENN DDT♀	
Larvae 25; adults 25	21 (17; 25)	21 (20; 22)	20 (15; 26)	23 (14; 27)
Larvae30; adults 25	20 (17; 24)	21 (20; 22)	19 (15; 25)	22 (19; 24)
Larvae 25; adults 30	18 (14; 21)	17 (15; 18)	22 (20; 23)	22 (19; 24)
Larvae 30; adults 30	17 (14; 21)	17 (15; 18)	22 (7; 22)	21 (17; 24)

95% confidence intervals (CI) are given

### The effect of elevated temperature on the expression of resistance phenotypes

**Fig. 3:**
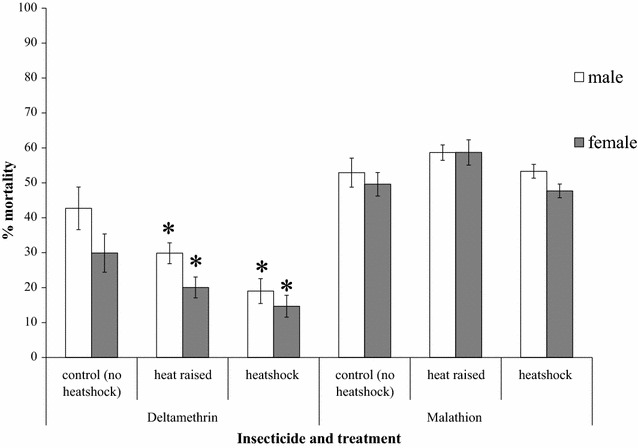
The effect of heat shock on the malathion- and deltamethrin-resistant phenotypes of insecticide-resistant *Anopheles arabiensis.* Adult SENN DDT individuals that emerged from larvae reared at a constant temperature of 30 °C showed a significant decrease in deltamethrin induced mortality compared to controls that had been reared at 25 °C and received no heat shock during their lifetime. Similarly, adults that were reared at 25 °C but received a heat shock prior to exposure (3-h heat shock at 37 °C with a 1-h recovery period) showed a similar decrease in deltamethrin-induced mortality. These findings were observed in both males and females. This effect was not observed for the malathion resistance phenotype. All exposures were performed under standard WHO bioassay conditions [26]

Rearing larvae at 30 °C or exposing them to 37 °C for 3 h elicited different responses in the malathion and deltamethrin resistance phenotypes of SENN DDT. Although neither the elevated larval-rearing temperature nor the short-term heat shock affected the malathion resistance phenotype (females: one-way ANOVA: p = 0.36, F = 2.34; males: one-way ANOVA: p = 0.26, F = 3.72), both treatments resulted in a marked decrease in deltamethrin-induced mortality (Fig. 3). This was true for both females (one-way ANOVA: p < 0.01; F = 70.5; df = 2) as well as males (one-way ANOVA p < 0.01; F = 35.3; df = 2).

**Fig. 4:**
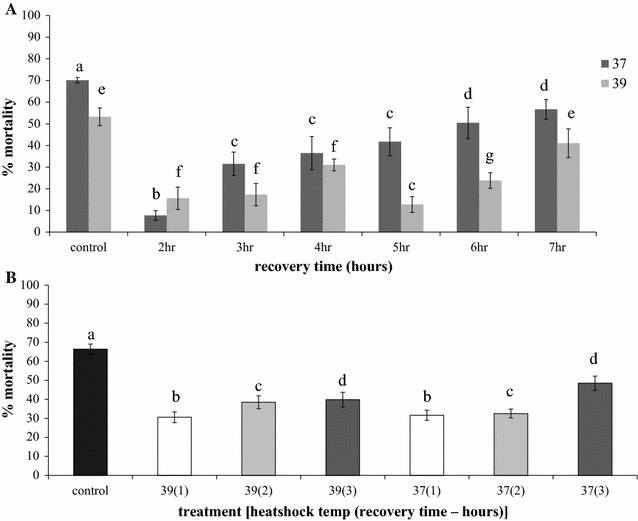
The progressive increase in mortality with time following the pyrethroid-resistance augmenting effects of heat shock in insecticide-resistant *Anopheles arabiensis.* Deltamethrin-induced mortality decreased after a short-term heat shock (37 °C for 3 h; 39 °C for 1 h, both with a 1-h recovery period) (**A**), as did λ-cyhalothrin induced mortality (**B**). Deltamethrin-induced mortality returned to pre-heat shock levels 7 h after the initial heat shock for the 39 °C heat shock but not the 37 °C heat shock treatment. The effects of the 37 °C heat shock decreased in a linear fashion (r^2^ = 0.95), while the linear decrease following exposure to the 39 °C heat shock was less pronounced (r^2^ = 0.63). There was no significant difference in the effect of 37 and 39 °C heat shock on λ-cyhalothrin resistance, and both showed a linear decrease in the expression of heat shock-induced resistance

It was also demonstrated that the effect of short-term heat shock was transient. When exposed to a short-term heat shock of 37 °C or a sub-lethal heat shock at 39 °C, deltamethrin-induced mortality decreased significantly, but mortality increased in the hours after recovery until reaching pre-heat shock levels 7 h after heat shock (one-way ANOVA: 37 °C—p < 0.01; F = 16, df = 6; 39 °C—p < 0.01, F = 10.9, df = 6) (Fig. 4A). The progressive increase in mortality with time was highly linear for the 37 °C treatment (r^2^ = 0.94), but less so for 39 °C (r^2^ = 0.63).

Deltamethrin resistance is not the only pyrethroid resistance phenotype that can be augmented by short-term heat shock. λ-cyhalothrin induced mortality was also significantly reduced by pre-exposure to 37 °C (one-way ANOVA: p < 0.01; F = 36.4, df = 3) and 39 °C (one-way ANOVA: p < 0.01; F = 29.2; df = 3). Both temperature treatments elicited the same level of resistance augmentation following insecticide induced mortality at 1, 2 and 3 h post exposure after the 37 and 39 °C treatments (one-way ANOVA: p = 0.12; F = 2.17, df = 5) (Fig. 4B).

**Fig. 5:**
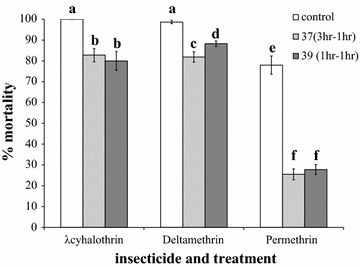
The effects of heat shock on the pyrethroid susceptibility of an insecticide susceptible *Anopheles arabiensis* strain. SENN is fully susceptible to λ-cyhalothrin and deltamethrin, but short-term heat shock resulted in a significant decrease in the mortality induced by these pyrethroids. Where low-level permethrin resistance exists, the effect is more marked. There was no significant difference in the reduction in permethrin- or λ-cyhalothrin-induced mortality after a 3-h heat shock at 37 °C or by a 39 °C shock for 1 h, both with a 1-h recovery period

Heat shock-induced reductions in pyrethroid susceptibility were also seen in the SENN strain. In Fig. 5 it is demonstrated that a 37 or a 39 °C shock caused a reduction in deltamethrin-induced mortality (one-way ANOVA: p = 0.01; F = 11.2; df = 2), λ-cyhalothrin-induced mortality (one-way ANOVA: p < 0.01; F = 15.8; df = 2) and permethrin-induced mortality (one-way ANOVA: p = 0.01; F = 20.9; df = 2). Both temperature treatments were equally efficient at reducing permethrin (two-sample t test: p = 0.55; t = −0.63) and λ-cyhalothrin (two-sample t test: p = 0.64; t = 0.47) susceptibilities, as there was no significant difference in mortality induced at either temperature for permethrin (two-sample t test: p = 0.55; t = −0.63) or λ-cyhalothrin (two-sample t test: p = 0.64; t = 0.47). The 37 °C heat shock, however, was more efficient at reducing deltamethrin susceptibility than the 39 °C heat shock (two-sample t test: p = 0.02; t = −2.40).

### The biological basis of heat shock-induced augmentation of pyrethroid resistance

Pyrethroid resistance is augmented by heat shock regardless of whether resistance mechanisms are absent (SENN) or present (SENN DDT). As SENN and SENN DDT are fixed as *kdr* SS and L1014F RR homozygotes, respectively, the effects of the mutation were not examined in this study. Two candidate explanations were sought. The first was that temperature elevation affected the detoxification enzyme systems of the mosquitoes involved. As the effects were more marked in SENN DDT, this strain was used to examine the underlying biological basis for these effects. An artificial in vitro study showed no significant differences in haem peroxidase (a proxy for cytochrome P450 activity) or general esterase activity (two-sample t test: cytochrome P450 p = 0.17; t = 1.37; α-esterase. p = 0.45, t = −0.76; β-esterase. p = 0.99, t = 0.02) when reactions were incubated at different temperatures. These results are summarized in Table 2. Furthermore, there was no significant difference in enzyme activity in non-heat-shocked adults compared to those recovering from heat shock in terms of P450 activity (one-way ANOVA: p = 0.2, F = 1.44, df = 7) or β-esterase activity (one-way ANOVA: p = 0.84; F = 0.49, df = 7). In contrast, although there was no difference in α-esterase activity of the recovering and heat-shocked specimens (one-way ANOVA: p = 0.23; F = 1.39), the activity of the non-heat-shocked control was significantly higher (one-way ANOVA: p < 0.01, F = 9.59, Tukey HSD critical Q = 4.3). These results are summarized in Table 3.

**Table 2:** Enzyme activities of the *Anopheles arabiensis* SENN DDT strain at periods following exposure to elevated temperatures

Treatment								
Enzyme	Control	2-h recovery	3-h recovery	4-h recovery	5-h recovery	6-h recovery	7-h recovery	8-h recovery
P450 (cytochrome P450 equivalents/mg/ml)	0.0009 (±0.001)	0.0013 (±0.001)	0.0014 (±0.001)	0.0014 (±0.001)	0.0015 (±0.001)	0.0015 (±0.001)	0.0012 (±0.001)	0.0014 (±0.001)
α-Esterase (α-Napathol equivalents/mg/ml)	0.0805 (±0.044)	0.0405 (±4.2 × 10^−3^)	0.0381 (±6.3 × 10^−3^)	0.0398 (±4.1 × 10^−3^)	0.0357 (±8.3 × 10^−3^)	0.0388 (±6.4 × 10^−3^)	0.0400 (±3.9 × 10^−3^)	0.0424 (±3.6 × 10^−3^)
β-Esterase (β-Napathol equivalents/mg/ml)	0.1279 (±0.086)	0.1198 (±0.022)	0.1139 (±0.028)	0.1173 (±0.011)	0.1051 (±0.0215)	0.1142 (±0.018)	0.1188 (±0.015)	0.1244 (±0.013)

Females of the resistant SENN DDT strain was allowed to recover for 2–8 h after a 1-h exposure to 39 °C. For each hour, individuals were collected and cold terminated, and the enzyme activity at that particular time point was assessed

**Table 3:** Enzyme activities of the *Anopheles arabiensis* SENN DDT strain at ambient (25 °C) and elevated (37 °C) temperatures

Treatment			Two-sample t test
Enzyme	Enzyme activity at 25 °C (±SD)	Enzyme activity at 37 °C (±SD)	
P450 (cytochrome P450 equivalents/mg/ml)	0.0016 (±0.001)	0.0012 (±0.001)	P = 0.19 T = 1.30; df = 94
α-Esterase (α-Napthol equivalents/mg/ml)	0.0805 (±0.006)	0.0895 (±0.010)	P = 0.45 T = −0.76; df = 94
β-Esterase (β-Napthol equivalents/mg/ml)	0.1279 (±0.012)	0.1283 (±0.023)	P = 0.98 T = −0.02; df = 94

**Fig. 6:**
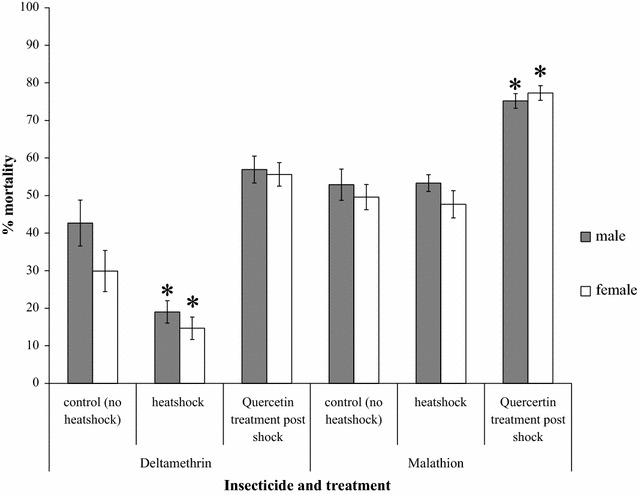
The role of heat shock proteins in the heat shock-induced effects on insecticide resistance phenotype of an insecticide-resistant *Anopheles arabiensis* strain. Treatment with the heat shock protein suppressing flavinoid quercetin returned deltamethrin-induced mortality levels to that of non-heat-shocked adults. Quercetin treatment of heat-shocked adults resulted in a significant increase in malathion-induced mortality

The second candidate explanation was that the induction of HSPs played a role in heat shock-induced pyrethroid resistance augmentation. This study demonstrated that the flavinoid quercetin could be delivered to mosquitoes via sugar supplementation without inducing increased mortality. Quercetin supplementation, and therefore inducible HSP inhibition, can return deltamethrin-induced mortality to pre-heat shock levels in the case of males and results in increased deltamethrin-induced mortality in females (Fig. 6). Quercetin-treated, heat-shocked adults did not differ in deltamethrin-induced mortality compared to untreated controls (two-sample t test: p = 0.49; t = −0.73), although heat-shocked adults that did not receive quercetin supplementation were significantly less likely to be killed by deltamethrin (two-sample t test: p < 0.01; t = 6.02). For malathion resistance, as previously demonstrated, heat shock did not result in a significant change in the resistance phenotype. After quercetin treatment, however, adults showed significantly higher levels of malathion-induced mortality than untreated controls (two-sample t test: p < 0.01, t = −4.6).

## Discussion

As with previous studies [23], no significant difference in larval development was observed between the two *A. arabiensis* strains differing in their insecticide resistance profiles. Increased temperature significantly decreased larval developmental time in both strains. The effects of fluctuating temperatures were not considered, although it should be noted that *A. arabiensis* copes particularly well with fluctuating temperatures [20]. The effect of fluctuating temperature on the differential development of resistant and susceptible *A. arabiensis* still needs to be quantified.

The effect of temperature on adult longevity was similar to previous studies [13]. Adults of the insecticide-resistant SENN DDT strain lived longer at elevated temperatures, regardless of the initial rearing temperature, than adults of the unselected, susceptible SENN strain. Furthermore, SENN adults showed a significantly reduced longevity at elevated temperatures, while the resistant SENN DDT adults did not show a change in longevity at elevated temperatures. These observations were consistent for both males and females. Together, these results suggest that elevated temperatures are advantageous for insecticide-resistant, rather than susceptible, *A. arabiensis* individuals because increased or uncompromised longevity at elevated temperatures is likely to yield greater fitness via increased fecundity.

Larval thermal cross-tolerance to insecticide intoxication has been demonstrated several times. In contrast to previous research, which focussed on short-term exposures to sub-lethal temperatures, this study demonstrated that a constant incubation of 30 °C could induce thermal cross-tolerance in adults [12]. It is also important to note that a previous study demonstrating adaptive thermotolerance raised the larvae of the experimental group at 30 °C before exposing them to a short-term heat shock [13], while the current study demonstrated that increasing the rearing temperature from 25 to 30 °C alone could induce similar changes in adult insecticide resistance expression.

Most examples of thermal cross-tolerance look only at the larval stage. In the current study, it was demonstrated that short-term heat exposure of adult mosquitoes is capable of inducing a similar augmentation of a resistance phenotype. Short-term exposure, however, had only a transient effect on the resistance phenotype, degrading within 8 h, unlike the sustained augmentation observed after increasing larval-rearing temperature. There are various examples of short-term exposure to elevated temperatures occurring in nature. The most important of these is the heat shock that occurs when a female takes a blood meal. However, further short-term environmental exposure to elevated temperatures (in particular, above the temperature that insectary-reared individuals would experience) could occur during the day. As reviewed by Glunt et al. [9], mean indoor temperatures in sub-Saharan Africa often exceed 27 °C during the day, possibly inducing heat shock effects in indoor-resting females. Therefore, although the short-term heat shock may degrade over the course of an evening, it is possible that this short heat exposure may affect the insecticide tolerance of indoor-resting females. It is noteworthy that, unlike previous studies [15], pyrethroid cross-resistance was evident while malathion cross-resistance was not induced.

Previous studies that have demonstrated thermal cross-tolerance have not mentioned the resistance status of the starting material [12, 31] or used only resistant individuals [15]. This present study demonstrated significant decreases in deltamethrin- and λ-cyhalothrin-induced mortality in individuals that were previously fully susceptible, and an even more marked decrease in mortality when low-level permethrin resistance was initially present. This highlights the importance of thermal exposure on subsequent susceptibility to insecticide intoxication.

Attempts have been made to explain the biochemical basis of these reactions. A previous study in *C. quinquefasciatus* demonstrated an increase in α-esterase activity after thermal exposure, but a decrease in β-esterase activity [15]. By contrast, in this study there is a significant decrease in α-esterase activity after thermal exposure, but no significant changes in β-esterase activity. As no definitive explanation could be derived from examination of detoxification enzyme activity, it was decided to examine another possible mechanism for heat-induced augmentation of pyrethroid resistance.

As propoxur application was able to induce HSP expression [12], the role of HSPs in the resistance phenotype was examined using quercetin synergism. Quercetin synergism negated the effects of heat shock-induced augmentation of the deltamethrin resistance phenotype. Furthermore, although heat shock did not significantly affect the malathion resistance phenotype and quercetin consumption alone did not significantly increase mortality, malathion-induced resistance was significantly increased after HSP synergism. This suggests a direct role for HSPs in the insecticide resistance phenotype.

There are several implications of the findings of this study. Previous work has raised concerns about the effect of climate change on vector control efforts [9]. A study in *Nilaparvata lugens* suggested that elevated temperatures associated with climate change could favour the survival of insecticide-resistant individuals [32]. This study highlights similar concerns. Previous studies have suggested that increasing environmental temperatures decrease the lifespan of the major malaria vector *A. gambiae* [17]. This is true for susceptible but not resistant *A. arabiensis* individuals. As heat shock augments pyrethroid resistance, this has implications for the dynamics of resistance management because of the increase in the expression of resistance intensity associated with exposure to increased heat. It can therefore be suggested, as in Ge et al. [32], that increasing environmental temperatures can favour insecticide-resistant populations, especially in insecticide-based control settings, possibly leading to significant epidemiological effects in terms of disease transmission.

Finally, data from this study implicate inducible HSPs as playing a vital role in the insecticide resistance phenotype. Previous studies have attempted to explain the resistance augmenting effect of blood feeding [24, 33, 34]. As it has been demonstrated that blood feeding increases HSP expression in both anophelines [35] and culicines [36], this may suggest a role for HSPs in blood meal-induced reduction of insecticide toxicity.

## Conclusion

It is concluded that exposure to thermal stress conditions increases the lifespan of insecticide-resistant *A. arabiensis*, but not their susceptible counterparts. Heat shock augments pyrethroid resistance where insecticide resistance mechanisms already exist, as well as resulting in a de novo resistance phenotype. Inducible HSPs likely play a role in this effect.

## References

[1] Berrigan D, Partridge L (1997). Influence of temperature and activity on the metabolic rate of adult *Drosophila melanogaster*. Comp Biochem Physiol A Physiol.

[2] Gillooly JF, Brown JH, West GB, Savage VM, Charnov EL (2001). Effects of size and temperature on metabolic rate. Science.

[3] Renault D, Bouchereau A, Delettre YR, Hervant F, Vernon P (2006). Changes in free amino acids in *Alphitobius diaperinus* (Coleoptera: Tenebrionidae) during thermal and food stress. Comp Biochem Physiol A Mol Integr Physiol.

[4] Feder ME, Hofmann GE (1999). Heat-shock proteins, molecular chaperones, and the stress response: evolutionary and ecological physiology. Annu Rev Physiol.

[5] Bayoh MN, Lindsay SW (2003). Effect of temperature on the development of the aquatic stages of *Anopheles gambiae* sensu stricto (Diptera: Culicidae). Bull Ent Res..

[6] Lyons CL, Coetzee M, Terblanche JS, Chown SL (2012). Thermal limits of wild and laboratory strains of two African malaria vector species, *Anopheles arabiensis* and *Anopheles funestus*. Malar J..

[7] Papanikolaou NE, Milonas PG, Kontodimas DC, Demiris N, Matsinos YG (2013). Temperature-dependent development, survival, longevity, and fecundity of *Propylea quatuordecimpunctata* (Coleoptera: Coccinellidae). Ann Entomol Soc Am.

[8] Muturi EJ, Nyakeriga A, Blackshear M (2012). Temperature-mediated differential expression of immune and stress-related genes in *Aedes aegypti* larvae. J Am Mosq Control Assoc..

[9] Glunt KD, Blanford JI, Paaijmans KP (2013). Chemicals, climate, and control: increasing the effectiveness of malaria vector control tools by considering relevant temperatures. PLoS Pathog.

[10] Hodjati MH, Curtis CF (1999). Effects of permethrin at different temperatures on pyrethroid-resistant and susceptible strains of *Anopheles*. Med Vet Entomol.

[11] Glunt KD, Paaijmans KP, Read AF, Thomas MB (2014). Environmental temperatures significantly change the impact of insecticides measured using WHOPES protocols. Malar J..

[12] Patil NS, Lole KS, Deobagkar DN (1996). Adaptive larval thermotolerance and induced cross-tolerance to propoxur insecticide in mosquitoes *Anopheles stephensi* and *Aedes aegypti*. Med Vet Entomol.

[13] Swain V, Seth RK, Mohanty SS, Raghavendra K (2008). Effect of temperature on development, eclosion, longevity and survivorship of malathion-resistant and malathion-susceptible strain of *Culex quinquefasciatus*. Parasitol Res.

[14] Swain V, Seth RK, Raghavendra K, Mohanty SS (2009). Impact of temperature on susceptible and resistant strains of *Culex quinquefasciatus* to synthetic pyrethroids. Acta Trop.

[15] Swain V, Seth RK, Raghavendra K, Mohanty SS (2009). Characterization of biochemical based insecticide resistance mechanism by thermal bioassay and the variation of esterase activity in *Culex quinquefasciatus*. Parasitol Res.

[16] Christiansen-Jucht C, Parham PE, Saddler A, Koella JC, Basanez MG (2014). Temperature during larval development and adult maintenance influences the survival of *Anopheles gambiae* s.s. Parasit Vectors..

[17] Christiansen-Jucht CD, Parham PE, Saddler A, Koella JC, Basanez MG (2015). Larval and adult environmental temperatures influence the adult reproductive traits of *Anopheles gambiae* s.s. Parasit Vectors..

[18] Coluzzi M, Sabatini A, Petrarca V, Di Deco MA (1979). Chromosomal differentiation and adaptation to human environments in the *Anopheles gambiae* complex. Trans R Soc Trop Med Hyg.

[19] Petrarca V, Vercruysse J, Coluzzi M (1987). Observations on the *Anopheles gambiae* complex in the Senegal River Basin, West Africa. Med Vet Entomol..

[20] Lyons CL, Coetzee M, Chown SL (2013). Stable and fluctuating temperature effects on the development rate and survival of two malaria vectors, *Anopheles arabiensis* and *Anopheles funestus*. Parasit Vectors..

[21] Kitau J, Oxborough RM, Tungu PK, Matowo J, Malima RC, Magesa SM (2012). Species shifts in the *Anopheles gambiae* complex: do LLINs successfully control *Anopheles arabiensis*?. PLoS ONE.

[22] Sharp BL, Le Sueur D, Bekker P (1990). Effect of DDT on survival and blood feeding success of *Anopheles arabiensis* in northern Kwazulu, Republic of South Africa. J Am Mosq Control Assoc..

[23] Oliver SV, Brooke BD (2013). The effect of larval nutritional deprivation on the life history and DDT resistance phenotype in laboratory strains of the malaria vector *Anopheles arabiensis*. Malar J..

[24] Oliver SV, Brooke BD (2014). The effect of multiple blood-feeding on the longevity and insecticide resistant phenotype in the major malaria vector *Anopheles arabiensis* (Diptera: Culicidae). Parasit Vectors..

[25] Hunt RH, Brooke BD, Pillay C, Koekemoer LL, Coetzee M (2005). Laboratory selection for and characteristics of pyrethroid resistance in the malaria vector *Anopheles funestus*. Med Vet Entomol.

[26] WHO. Test procedures for insecticide resistance monitoring in malaria vector mosquitoes. Geneva: World Health Organization. 2013. http://www.whoint/malaria/publications/atoz/9789241505154/en/index.html. Accessed 26 Jan 2017.

[27] Brogdon WG, McAllister J (2004). Insecticide resistance and vector control. J Agromed..

[28] Brogdon WG, McAllister JC, Vulule J (1997). Heme peroxidase activity measured in single mosquitoes identifies individuals expressing an elevated oxidase for insecticide resistance. J Am Mosq Control Assoc..

[29] Penilla RP, Rodríguez AD, Hemingway J, Torres JL, Arredondo-Jiménez JI, Rodríguez MH (1998). Resistance management strategies in malaria vector mosquito control. Baseline data for a large-scale field trial against *Anopheles albimanus* in Mexico. Med Vet Entomol.

[30] Gupta SC, Siddique HR, Mathur N, Vishwakarma AL, Mishra RK, Saxena DK, Chowdhuri DK (2007). Induction of hsp70, alterations in oxidative stress markers and apoptosis against dichlorvos exposure in transgenic *Drosophila melanogaster*: modulation by reactive oxygen species. Biochim Biophys Acta.

[31] Raghavendra K, Barik TK, Adak T (2010). Development of larval thermotolerance and its impact on adult susceptibility to malathion insecticide and *Plasmodium vivax* infection in *Anopheles stephensi*. Parasitol Res.

[32] Ge LQ, Huang LJ, Yang GQ, Song QS, Stanley D, Gurr GM, Wu JC (2013). Molecular basis for insecticide-enhanced thermotolerance in the brown planthopper *Nilaparvata lugens* Stal (Hemiptera:Delphacidae). Mol Ecol.

[33] Halliday WR, Feyereisen R (1987). Why does DDT toxicity change after a blood meal in adult female *Culex pipiens*?. Pest Biochem Physiol..

[34] Oliver SV, Brooke BD (2016). The role of oxidative stress in the longevity and insecticide resistance phenotype of the major malaria vectors *Anopheles arabiensis* and *Anopheles funestus*. PLoS ONE.

[35] Benoit JB, Lopez-Martinez G, Patrick KR, Phillips ZP, Krause TB, Denlinger DL (2011). Drinking a hot blood meal elicits a protective heat shock response in mosquitoes. Proc Natl Acad Sci USA.

[36] Lahondere C, Lazzari CR. Thermal stress and thermoregulation during feeding in mosquitoes. In: Manguin S, editors. New insights into malaria vectors. InTech. doi: 10.5772/56288. 2013. http://www.intechopen.com/books/anopheles-mosquitoes-new-insights-into-malaria-vectors/thermal-stress-and-thermoregulation-during-feeding-in-mosquitoes. Accessed 26 Jan 2017.

